# *SiFTL* and *SiHd3a* are positive regulators of flowering time in sesame (*Sesamum indicum* L.)

**DOI:** 10.3389/fpls.2025.1716212

**Published:** 2025-12-03

**Authors:** Fengli Zhao, Zhenwei Du, Chengqi Cui, Ke Wu, Xiaolin Jiang, Jingjing Wang, Ruping Zhang, Yadong Fan, Robert J. Henry, Yanyang Liu, Hongxian Mei, Haiyang Zhang

**Affiliations:** 1 Henan Sesame Research Center, Henan Academy of Agricultural Sciences, Zhengzhou, China; 2 The Shennong Laboratory, Zhengzhou, China; 3 Queensland Alliance for Agriculture and Food Innovation, University of Queensland, Brisbane, QLD, Australia

**Keywords:** sesame, flowering time, linkage mapping, flowering locus T like, heading date 3a

## Abstract

Flowering time, a critical trait for crop adaptation, is regulated by plants’ ability to sense light signals, which influences gene expression and the transition from vegetative to reproductive growth. In this study, five populations of sesame (*Sesamum indicum*) were developed by crossing ‘Yuzhi 4’ with ‘BS377’ (late-flowering variety). Linkage mapping analysis revealed that a ~400 kb region on chromosome 11 had the most significant and stable effect on flowering time. *FLOWERING LOCUS T-like* (*FTL*) and *HEADING DATE 3A* (*Hd3a*), located within the candidate region, are associated with flowering time as revealed by RNA-seq and qRT-PCR analyses. Integrated analyses of *SiFTL* gene structure and transcript level revealed that the absence of *SiFTL* expression leads to the late-flowering phenotype in BS377. Moreover, *SiFTL* and *SiHd3a*, which are highly homologous to *Arabidopsis FT* and *TWIN SISTER OF FT* (*TSF*), are associated with earlier flowering. Furthermore, the Yuzhi 4 haplotypes of *SiFTL* and *SiHd3a* locus co-segregated with the early-flowering trait in a recombinant inbred line (RIL) population. In addition, heterologous expression of the Yuzhi 4 haplotypes of *SiFTL* and *SiHd3a* significantly accelerated flowering in *Arabidopsis* Col-0 and the *ft-10* mutant under both long-day (LD) and short-day (SD) conditions. These haplotypes may function as universal floral activators downstream of flowering pathways. These findings not only enhance our understanding of the molecular mechanisms controlling flowering time in sesame but also offer valuable genetic resources for breeding early-maturing varieties.

## Introduction

Flowering marks the transition from vegetative growth to reproductive development in plants, reflecting their adaptation to local seasonal and environmental conditions (Kloosterman et al., 2012). Flowering time impacts biomass and yield in many crops, making it a key target for crop breeding (Schiessl et al., 2014; Ortega et al., 2019; Li et al., 2021). In *Arabidopsis*, flowering time is regulated by multiple interconnected genetic pathways, including the photoperiod, autonomous, vernalization, gibberellic acid, and ambient temperature pathways (Jin et al., 2015; Huang et al., 2017; Wang et al., 2024). *FLOWERING LOCUS T* (*FT*) a central component of these pathways, integrates signals from various pathways to promote flowering (Lifschitz et al., 2006; Song et al., 2013; Mäckelmann et al., 2024). The FT protein, a member of the phosphatidylethanolamine-binding protein (PEBP) family, is highly conserved across plant species and plays critical roles in growth and differentiation signaling pathways (Lifschitz et al., 2006). In *Arabidopsis*, the PEBP gene family is divided into three main subfamilies: *FT-like*, *TERMINAL FLOWER1* (*TFL1*)*-like*, and *MOTHER OF FT AND TFL1* (*MFT*)*-like* (Chardon and Damerval, 2005). The *FT-like* clade comprises *FT* and *TWIN SISTER OF FT* (*TSF*), which act as potent floral activators (Jin et al., 2015).The *TFL1-like* subfamily consists of *TFL1*, *ARABIDOPSIS THALIANA CENTRORADIALIS* (*ATC*), and *BROTHER OF FT AND TFL1* (*BFT*), are key repressors that modulate flowering time and inflorescence architecture (Chardon and Damerval, 2005). The *MFT-like* subfamily contains only *MFT*, which functions in seed development and germination during both gametophyte and sporophyte stages (Dong et al., 2020).

In various plants, such as *Arabidopsis* and rice, the FT protein is primarily synthesized in the leaves (specifically in phloem companion cells) and transported through the phloem to the nuclei of shoot apical meristem cells (Neumaier and James, 1993; Tamaki et al., 2007). There, it physically interacts with 14-3–3 proteins and the basic leucine zipper transcription factor FLOWERING LOCUS D (FD) to form the florigen activation complex (Corbesier et al., 2007; Taoka et al., 2011). This complex directly binds to the promoters of floral meristem identity genes, such as *LEAFY* and *APETALA1*, activating their expression to initiate floral differentiation (Kobayashi et al., 2012). Heterologous expression of *FT* orthologs from various plant species has been shown to induce flowering, demonstrating the highly conserved function of *FT* across both dicots and monocots, including rice, wheat, *Arabidopsis*, tomato, soybean, *Populus*, and others (Kojima et al., 2002; Lifschitz et al., 2006; Corbesier et al., 2007; Komiya et al., 2008; Kong et al., 2010; Hsu et al., 2011; Rinne et al., 2011; Wang et al., 2020; Gu et al., 2022). Although the genetic basis of flowering time has been extensively studied in model plants, the genetic regulators of this trait in sesame, particularly the roles of flowering time gene, have not been fully elucidated.

Sesame (*Sesamum indicum L.*), a member of the Pedaliaceae family, is a significant oilseed crop renowned for its edible seeds and high-quality oil. It is widely cultivated in tropical and subtropical regions worldwide (Miao et al., 2021). Sesame is a short-day (SD) plant, typically delaying flowering and extending its vegetative growth phase under long-day (LD) conditions, resulting in late maturity (Suddihiyam et al., 1992; Zhou et al., 2018). Conversely, under SD conditions, sesame flowers earlier and completes its life cycle more quickly (Rhind, 1935; Ghosh, 1955). Flowering time is a critical trait that significantly impacts sesame yield and adaptation (Sinha et al., 1973; Kumazaki et al., 2008, 2009; Miao et al., 2021; Sabag et al., 2024). Phylogenetic analysis of *CONSTANS-like* (*COL*) genes suggests that *SiCOL1* likely plays a role in regulating flowering in sesame, and natural variations in *SiCOL1* may be associated with the expansion of sesame cultivation to high-latitude regions (Zhou et al., 2018). Expression profiles of *FT* and circadian clock-associated genes in early- and late-flowering sesame varieties indicate that *SiCOLs* or their regulators may be the causal genes influencing flowering time variation (López et al., 2023). Additionally, a key single nucleotide polymorphism (SNP) site (C allele) is found in the *FLOWERING LOCUS T-like* (*FTL*) gene, which significantly shortens flowering time under suitable sowing conditions (Sabag et al., 2024). However, there is limited information available on how genes regulate flowering time in sesame.

To uncover the molecular mechanisms controlling flowering in sesame, we selected two sesame germplasms: Yuzhi 4, a high agronomic performance and broad adaptability, and BS377, a late-flowering variety with small seed size and high lignan content, which exhibits prolonged vegetative growth. We crossed these two varieties and recorded flowering time across five generations (BC_1_, BC_1_F_2_, F_2_, F_2:3_, and recombinant inbred line (RIL) progenies). Using linkage mapping and RNA sequencing (RNA-seq), we identified potential flowering time regulators, pinpointing candidate genes *SiFTL* and *HEADING DATE 3A* (*Hd3a*) within a ~400 kb region on chromosome 11 associated with flowering time. Our findings provide insights into genetic mechanisms controlling flowering time in sesame, offering a foundation for future germplasm improvement strategies to optimize early maturation and environmental adaptability.

## Materials and methods

### Plant materials and growth conditions

**Figure 1:**
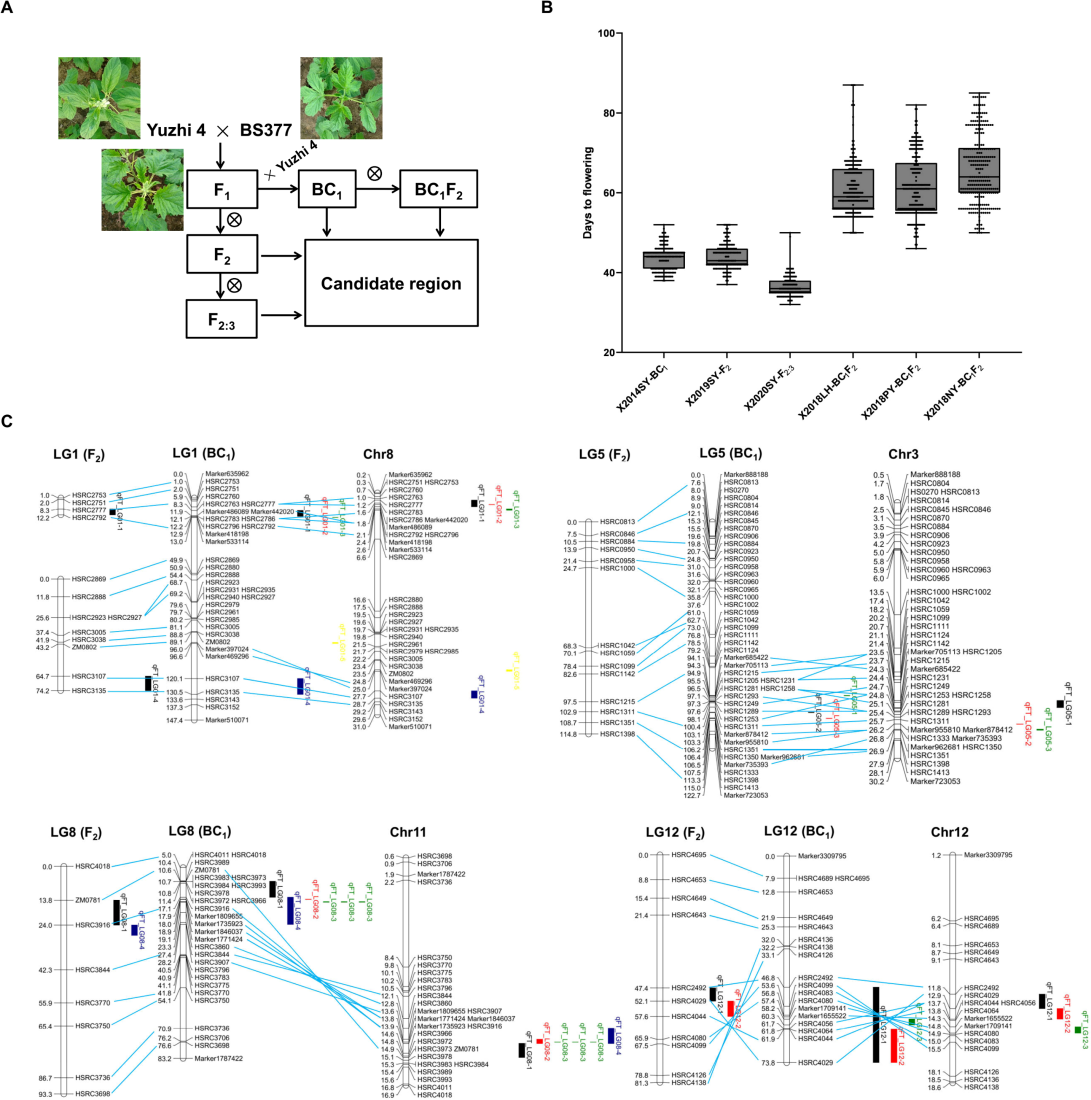
QTL mapping of flowering time in sesame. **(A)** Segregating populations generated by crossing the photoperiod-insensitive variety Yuzhi 4 with the photoperiod-sensitive variety BS377. Backcrossing used Yuzhi 4. **(B)** Flowering time of four populations in different environments. SY, LH, PY and NY represent Sanya, Luohe, Pingyu, and Nanyang. **(C)** QTL of flowering time identified in different populations.

The seeds of Yuzhi 4 and BS377 sesame varieties were obtained from the sesame germplasm repository at the Henan Academy of Agricultural Sciences in Zhengzhou, Henan Province, China. Yuzhi 4 is an elite cultivar released in the 1980s and has been widely cultivated in most sesame-growing regions in China over the past three decades due to its high agronomic performance and broad adaptability. BS377, an unnamed cultivar introduced from Bangladesh, exhibits a long vegetative growth period or even fails to flower under natural conditions in temperate regions due to its photoperiod sensitivity. Two populations, F_2_ and BC_1_, were developed from a cross between Yuzhi 4 (as the maternal and recurrent parent) and BS377 (as the paternal parent). These populations were self-pollinated to produce F_2:3_ and BC_1_F_2_ families, respectively (**Figure 1**). Phenotypic data were collected in Sanya (N18°14’, E109°29’; hereafter SY), Pingyu (N32°59’, E114°42’; hereafter PY), Nanyang (N32°54’, E112°24’; hereafter NY), and Luohe (N33°37’, E113°58’; hereafter LH). The day length in the summer (from 5–7 th May to 22–24 th July) of PY, NY and LH, ranged from 13.6 h to 14.3 h, whereas in the winter (from 7–8 th November to 20–21 th January) in SY, daylight was sustained for from 11.0 h to 11.3 h.

### Plant samples and treatments

The two parents and ~ 150 plants were randomly selected from the F_2_ and BC_1_ populations for genotyping and linkage map construction. For phenotypic data collection in field trials, 150 BC_1_ and 146 F_2_ individuals, as well as 128 F_2:3_ and 150 BC_1_F_2_ families, were utilized. The BC_1_F_2_ families, each with two replicates, were planted in LH, PY and NY. Flowering time was measured as the number of days from sowing to when 60% of the plants were flowering (approximately 7 out of 12 individual plants). The determination of flowering date was based on the opening of the first flower on a single plant of BC_1_ and F_2_, and the opening of the first flower on more than 60% of the plants of BC_1_F_2_, F_2:3_ and RIL populations (**Supplementary Table S1**).

**Figure 2:**
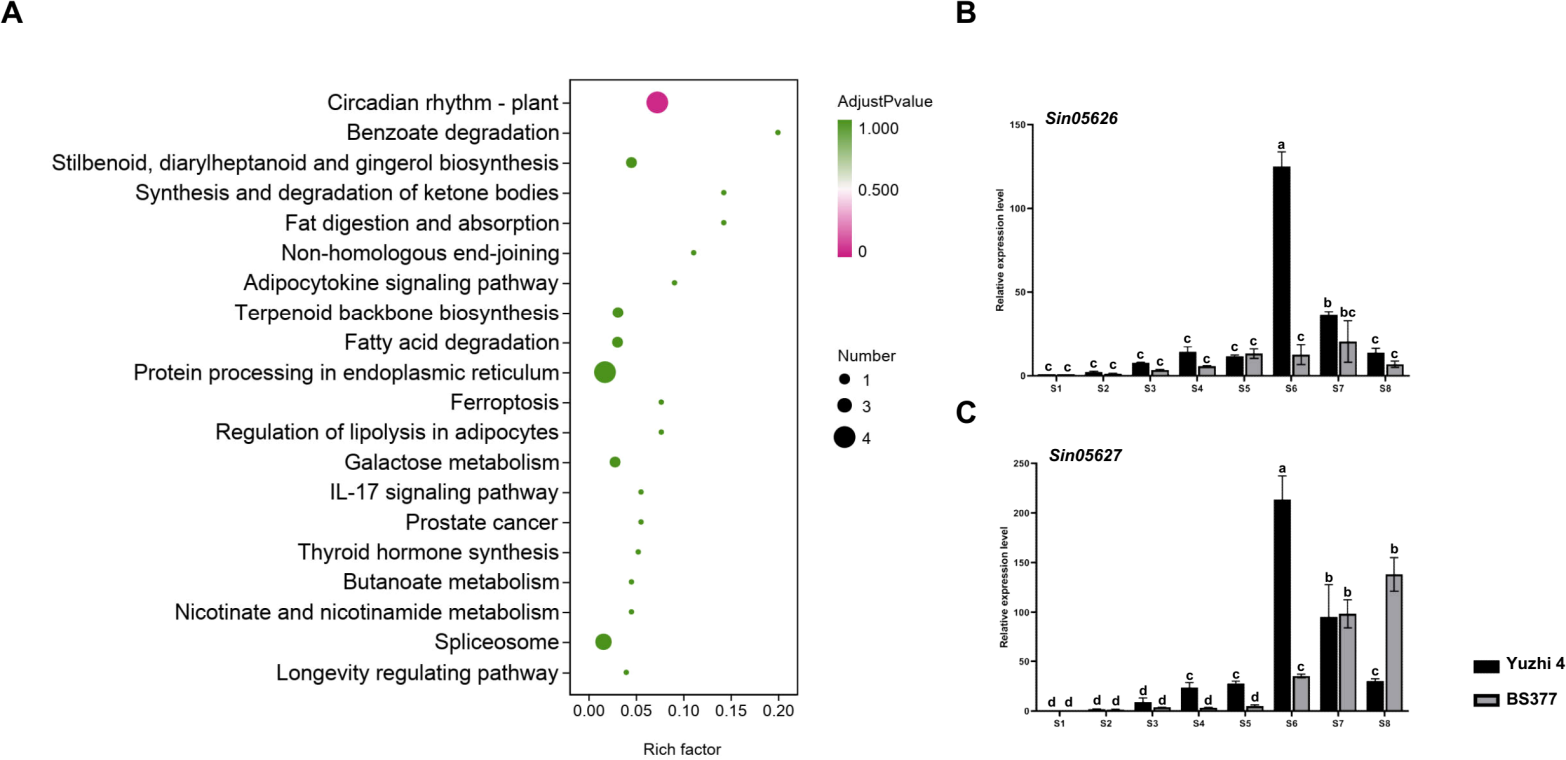
The DEGs in candidate region revealed by RNA-seq and qRT-PCR. **(A)** KEGG pathway enrichment analysis for genes in the candidate regions based on RNA-seq data. **(B)** qRT-PCR analysis of *Sin05626* during eight developmental stages of Yuzhi 4 and BS377 new leaves. **(C)** qRT-PCR analysis of *Sin05627* during eight developmental stages of Yuzhi 4 and BS377 new leaves. S1, S2, S3, S4, S5, S6, S7, S8 represent the leaves of the Yuzhi 4 variety at 5, 10, 15, 20, 25, 30, 35 and 40 days after sowing. S1, S2, S3, S4, S5, S6, S7, S8 represent the leaves of the BS377 variety at 5, 15, 35, 60, 65, 70, 75, and 90 d after sowing. S6 and S7 indicate the pre-budding and post-budding stages, respectively, while S8 denotes the post-flowering stage. Different letters indicate the statistical difference among samples at P ≤ 0.05 according to Duncan’s multiple range test.

Leaves from the Yuzhi 4 variety were collected at eight developmental stages (S1, S2, S3, S4, S5, S6, S7, and S8), corresponding to 5, 10, 15, 20, 25, 30, 35, and 40 days (d) after sowing (**Figure 2**; **Supplementary Figure 1**). Leaves of the late-flowering BS377 variety were collected at the corresponding developmental stages as Yuzhi 4, specifically at 5, 15, 35, 60, 65, 70, 75, and 90 d after sowing. S6 and S7 indicate the pre-budding and post-budding stages, respectively, while S8 denotes the post-flowering stage. Sampling was conducted at 9:00 am in a controlled phytotron under a 12 h light (8:00 to 20:00) and 12 h dark cycle. Samples from each stage were ground into powder using liquid nitrogen and stored at - 80 °C for subsequent experiments.

### Linkage map construction and quantitative trait loci mapping

In a previously study, we constructed a genetic map with 351 SSRs and 3,548 specific-locus amplified fragments (SLAFs) for the BC_1_ population (Mei et al., 2023; **Supplementary Table S2**). The F_2_ population was genotyped using a subset of 166 SSR markers, including 152 markers evenly distributed across the BC_1_-derived genetic map and 14 dominant markers (containing Yuzhi 4 alleles) excluded from the BC_1_ analyses (Mei et al., 2023; **Supplementary Table S2**). The genetic map was constructed using JoinMap 4.0. QTL mapping was performed using the IciMapping 4.1 software (Meng et al., 2015) with the inclusive composite interval mapping additive (ICIM-ADD) model (Li et al., 2007), and the significant LOD threshold was determined by 1,000 permutations (P = 0.05). Phenotypic variation explained (PVE) by each QTL was calculated based on variance contribution. Genetic maps and QTLs were visualized using MapChart 2.3 (Voorrips, 2002).

### Expression analysis

Total RNA was extracted from eight developmental stages of Yuzhi 4 and BS377 using an EZNA Plant RNA Kit (R6827-01, Omega Biotek, USA) following the manufacturer’s instructions. Total RNA was used for library construction and subjected to deep sequencing on an Illumina novaseq 6000 (Kindstar Sequenon Biotechnology (Wuhan) Co., LTD). Raw paired-end sequencing reads were quality controlled and filtered using fastp (version 0.23.4) (Chen et al., 2018). Reads shorter than 15 base pairs were removed, and bases with a Phred quality score below 20 were trimmed. The high-quality clean reads data were aligned to the sesame reference genome Yuzhi 4, and gene expression levels were quantified using transcripts per million (TPM). Gene annotations were performed by aligning BLAST search results to multiple databases, including Gene Ontology (GO), Kyoto Encyclopedia of Genes and Genomes (KEGG), COG/KOG, Pfam, NCBI non-redundant protein sequences (Nr), and the manually curated UniProt Knowledgebase, providing functional information for gene characterization. The RNA-seq data are available from BIG Submission (GSA accession: CRA032111). The candidate genes within the region were functionally annotated using KEGG and GO analyses, and their expression levels were quantified using TPM analysis.

To validate the RNA-seq data, approximately 1.5 mg of total RNA was reverse-transcribed into cDNA using the MightyScript First Strand cDNA Synthesis Master Mix (Sangon biotech, China). The quantitative real-time polymerase chain reaction (qRT-PCR) primers were designed using Vector NTI, avoiding conserved regions, with amplicon lengths ranging from 120–160 bp (**Supplementary Table S3**). The qRT-PCR reactions were performed using Taq Pro Universal SYBR qPCR Master Mix (Nanjing, China) with the following parameters on the LightCycler480 system. The relative gene expression levels were determined using the comparative 2^-ΔΔCT^ method (Livak and Schmittgen, 2001; **Figure 2**). Gene expression levels were normalized using the sesame housekeeping gene *SiTUB* (Wei et al., 2013). All experiments were performed in triplicate using biological replicates.

### Gene structure and phylogenetic analysis

**Figure 3:**
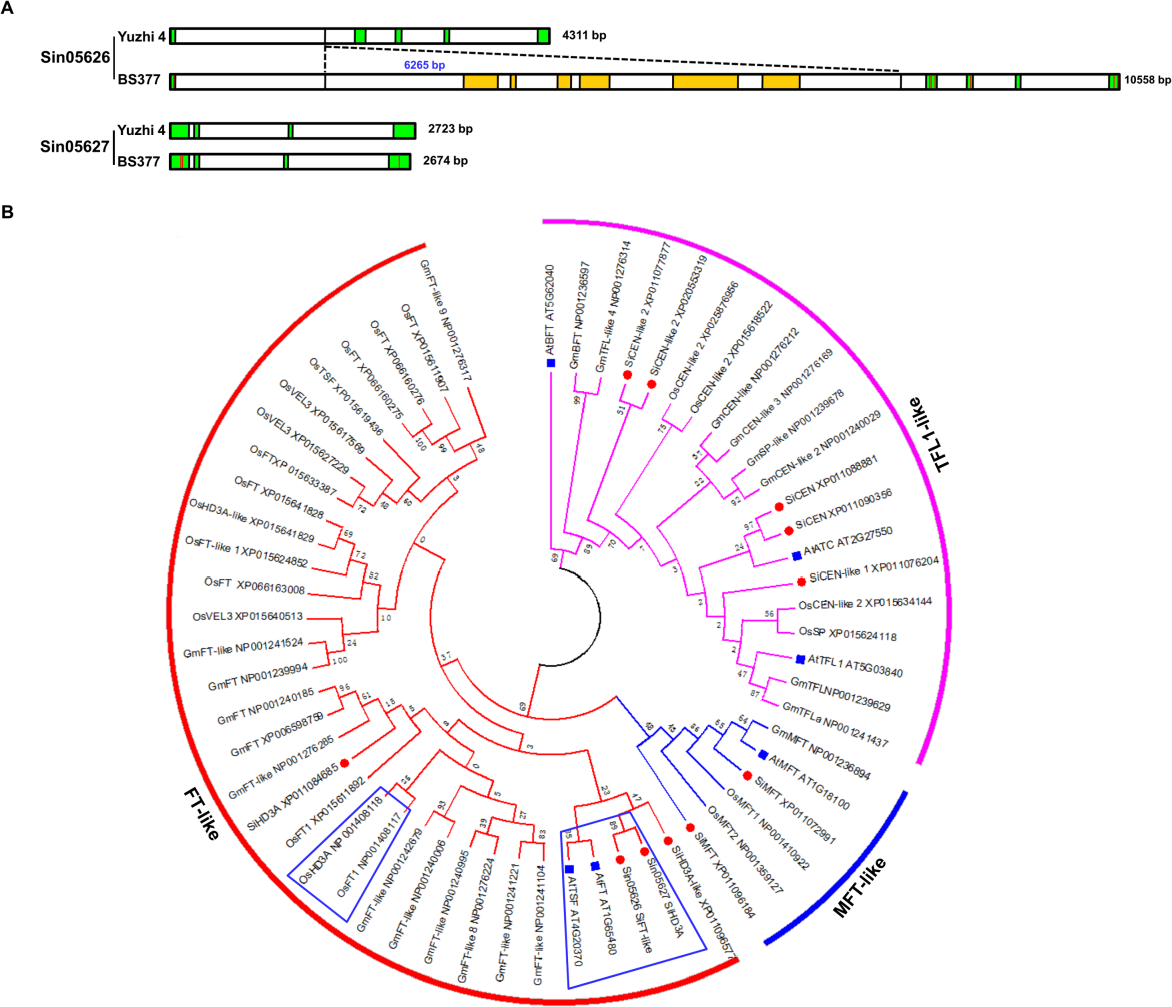
Gene structure and phylogenetic relationship analysis for candidate genes. **(A)** Gene structure analysis of *Sin05626* and *Sin05627* comparing the sequences from Yuzhi 4 and BS377 genome. Green rectangle represents exon, white rectangle represents intron, yellow rectangle represents conserved domain, red line represents SNP in the exon. **(B)** Phylogenetic relationship of *PEBP* family across various species containing *Arabidopsis* (blue), rice, soybean, and sesame (red), constructed using the neighbor-joining method in MEGA5 software. Proteins SiFTL and SiHd3a in blue box.

The genomes of Yuzhi 4 and BS377 were aligned using SyRI software, although their genome sequences have not yet been released (**Figure 3**; **Supplementary Figure 2**). For homologous gene identification, the *Arabidopsis* PEBP amino acid sequences from TAIR (The *Arabidopsis* Information Resource) were used as queries to search the NCBI non-redundant protein database (nr) using BLAST-P (Altschul et al., 1990), and removed redundant genes from the same clade (100% similarity). Following sequence and conserved domain validation, full-length PEBP amino acid sequences from *Oryza sativa*, *Glycine max*, and *Sesamum indicum* (https://www.ncbi.nlm.nih.gov/datasets/genome/GCF_000512975.1/) were used to construct a phylogenetic tree. The tree was generated using the neighbor-joining method in MEGA 5.0, with the p-distance model, complete gap deletion, and 1000 bootstrap replicates (Zhao et al., 2018; **Figure 3**).

### Haplotypes of *SiFTL* and *SiHd3a* is associated with flowering time

**Figure 4:**
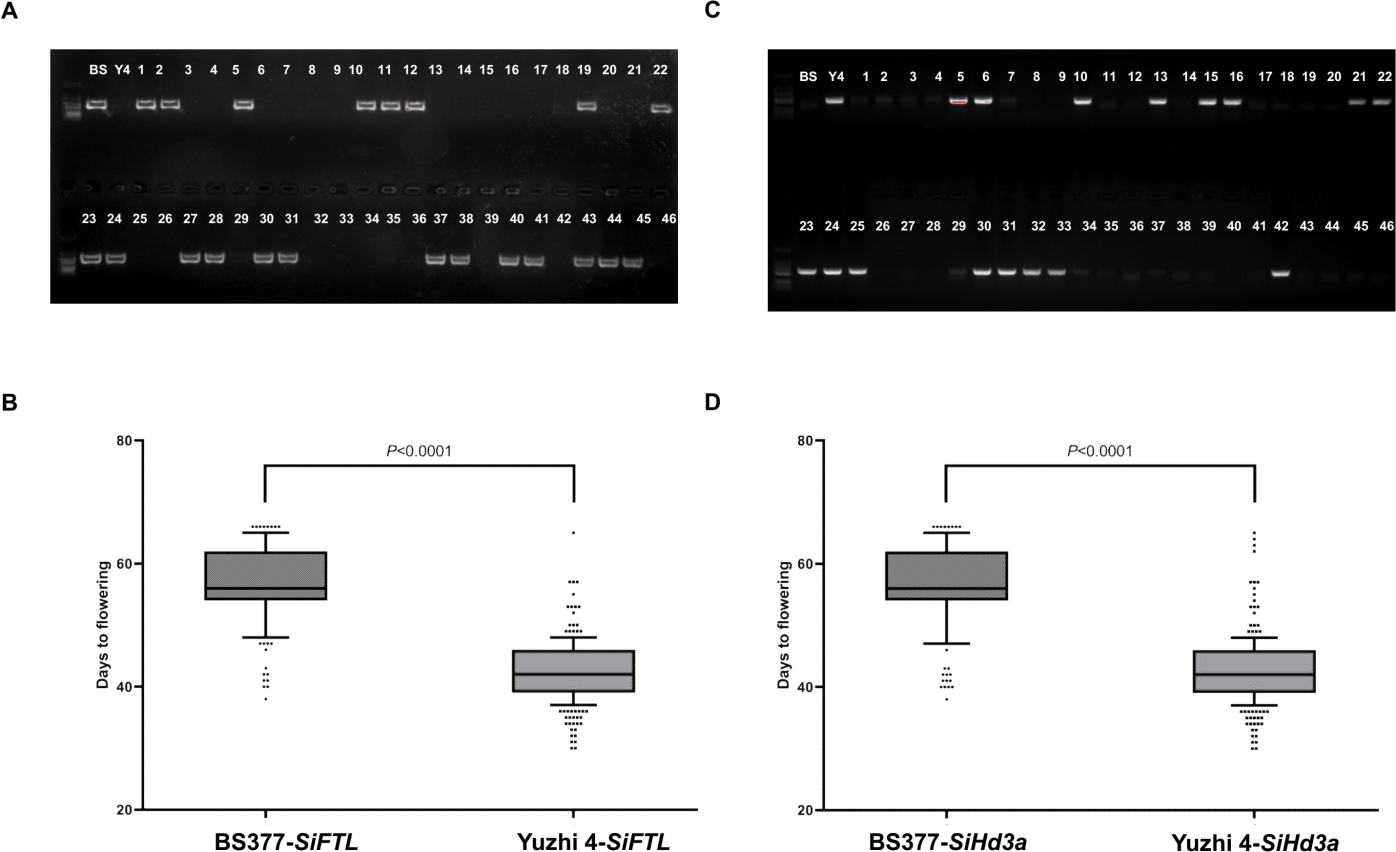
Validation *SiFTL* and *SiHd3a* alleles for flowering time under Yuzhi 4 and BS377 backgrounds using a RIL population. **(A)** Agarose gel electrophoresis detected BS377 haplotype of *SiFTL* in RIL populations. **(B)** Validation of *SiFTL* haplotype on chromosome 11 for flowering time. **(C)** Agarose gel electrophoresis detected Yuzhi 4 haplotype of *SiHd3a* in RIL populations. **(D)** Validation of *SiHd3a* haplotype on chromosome 11 for flowering time. The statistical difference among samples at P ≤ 0.001 according to Duncan’s multiple range test.

The RIL population was developed by crossing Yuzhi 4 with BS377 and eight generations of selfing, producing a phenotypically stable population. To evaluate the relationship among annotation indicated that *SiFTL*, *SiHd3a*, and flowering time, a RIL population (n = 425) was planted in PY on August 1, 2024, and genomic DNA was extracted for analysis (**Figure 4**; **Supplementary Table S1**). Primers were designed based on sequence differences between BS377 and Yuzhi 4 (**Figure 4**; **Supplementary Figure 3**; **Supplementary Table S3**). The *SiFTL* band presence corresponds to the BS377 haplotype, while the *SiHd3a* band corresponds to the Yuzhi 4 haplotype (**Figures 4A, C**). PCR amplification experiments were performed with these primers, and their specific amplification of target fragments was confirmed via electrophoresis.

### Generation of transgenic plants

**Figure 5:**
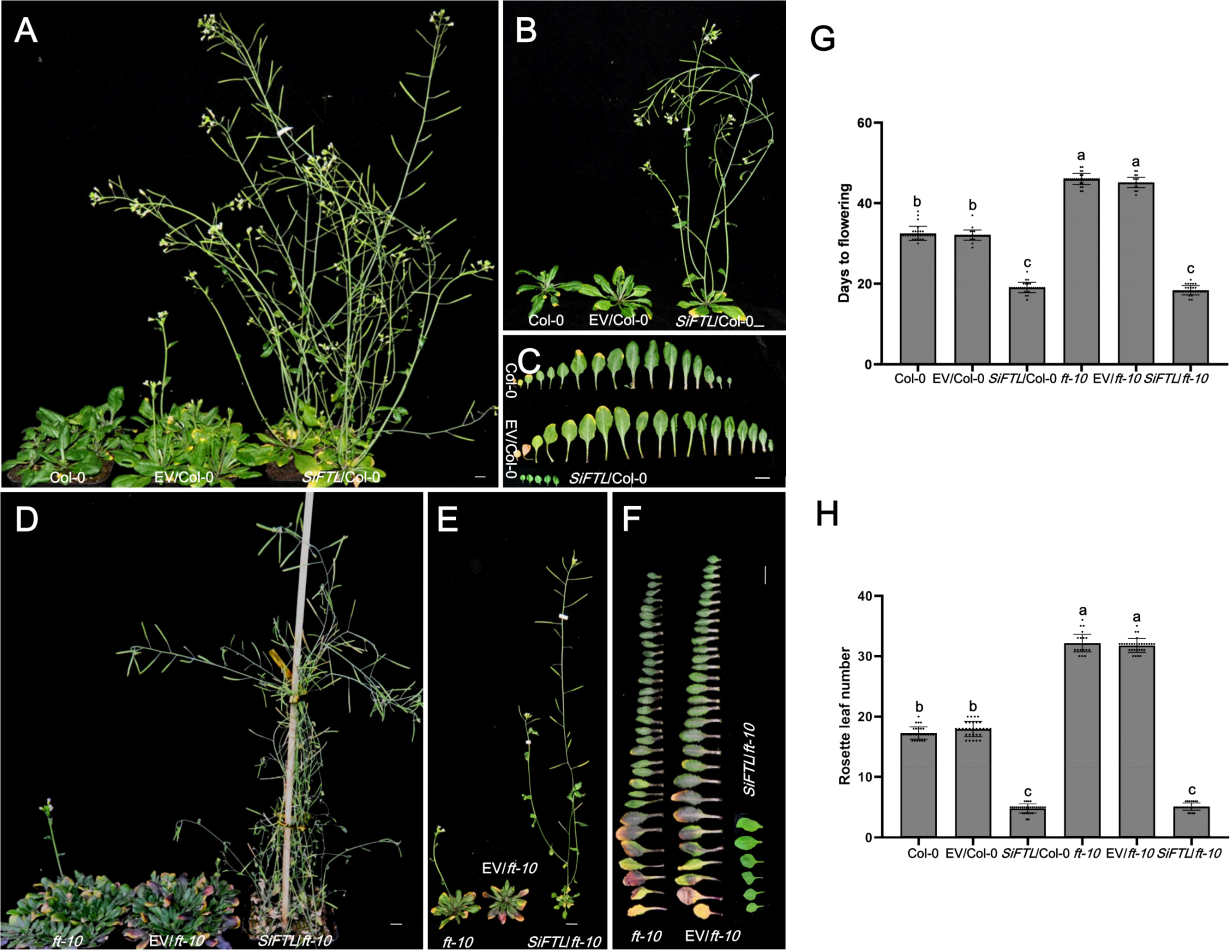
Days to flowering of transgenic *Arabidopsis* with overexpressed *SiFTL* under LD condition. **(A, B)** Photographs of 37 d after sowing Col-0 and *SiFTL* transgenic lines. **(C)** Photographs of flowering stages Col-0 and *SiFTL* transgenic lines leaves. **(D, E)** Photographs of 51 d after sowing *ft-10* mutant and *SiFTL* transgenic lines. **(F)** Photographs of flowering stages *ft-10* mutant and *SiFTL* transgenic lines leaves. **(G)** Days to flowering of flowering stages Col-0, *ft-10* mutant and *SiFTL* transgenic lines. **(H)** Rosette leaf number of flowering stages Col-0, *ft-10* mutant and *SiFTL* transgenic lines. Col-0 represents the wild-type *Arabidopsis* Col-0, EV/Col-0 represents overexpressed empty vector C15 in *Arabidopsis* Col-0, *SiFTL*/Col-0 represents overexpressed *SiFTL* in *Arabidopsis* Col-0. *ft-10* represents the *Arabidopsis ft-10* mutant, EV/*ft-10* represents overexpressed empty vector C15 in *ft-10* mutant, *SiFTL*/*ft-10* represents over expressed *SiFTL* in *ft-10* mutant. Values followed by different letters in a column are significantly different at P ≤ 0.05 according to Duncan’s multiple range tests. Bars =1 cm.

**Figure 6:**
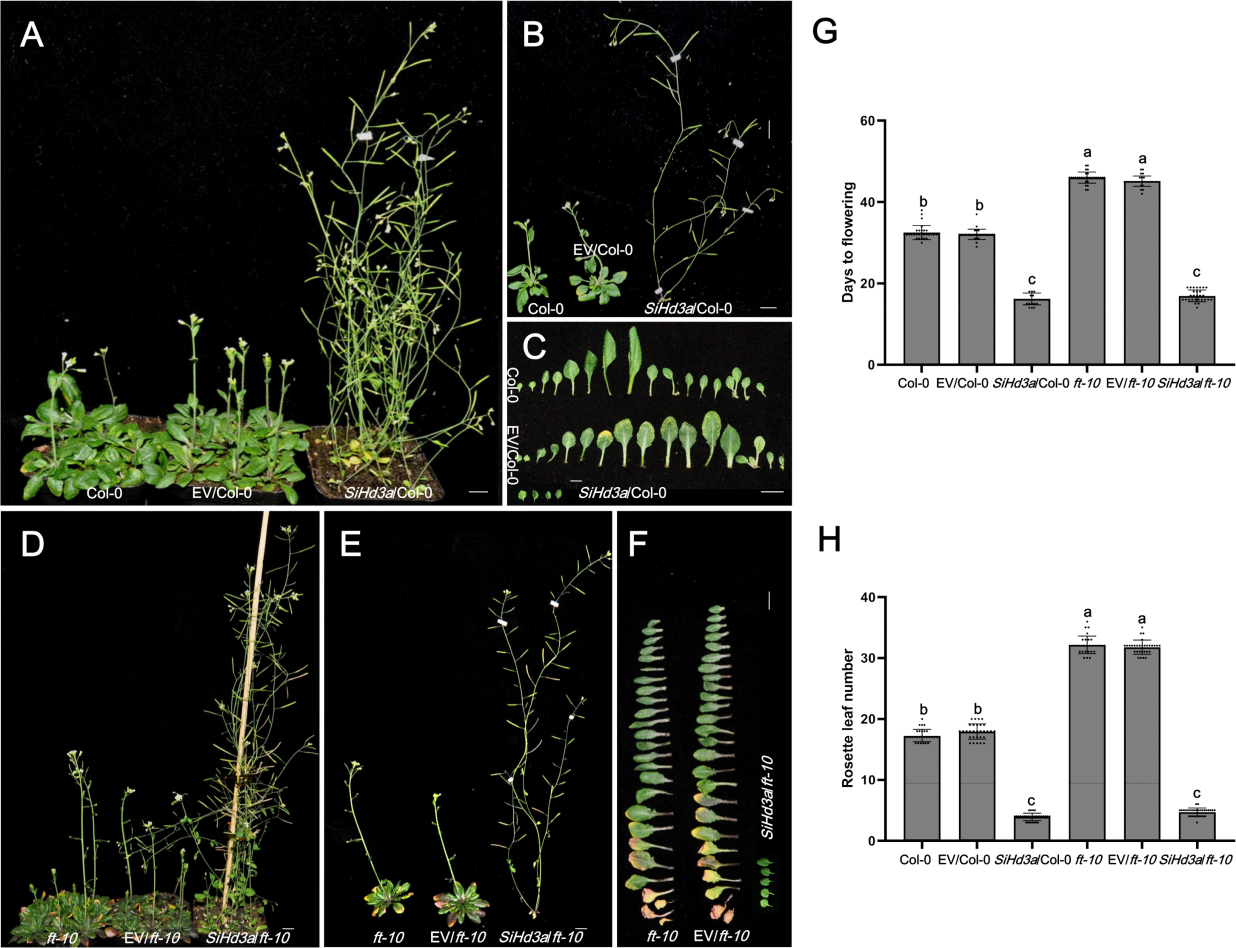
Days to flowering of transgenic *Arabidopsis* with overexpressed *SiHd3a* under LD condition. **(A, B)** Photographs of 37 d after sowing Col-0 and *SiHd3a* transgenic lines. **(C)** Photographs of flowering stages Col-0 and *SiHd3a* transgenic lines leaves. **(D, E)** Photographs of 51 d after sowing *ft-10* mutant and *SiHd3a* transgenic lines. **(F)** Photographs of flowering stages *ft-10* mutant and *SiHd3a* transgenic lines leaves. **(G)** Days to flowering of flowering stages Col-0, *ft-10* mutant and *SiHd3a* transgenic lines. **(H)** Rosette leaf number of flowering stages Col-0, *ft-10* mutant and *SiHd3a* transgenic lines. Col-0 represents the wild-type *Arabidopsis* Col-0, EV/Col-0 represents overexpressed empty vector C15 in *Arabidopsis* Col-0, *SiHd3a*/Col-0 represents overexpressed *SiHd3a* in *Arabidopsis* Col-0. *ft-10* represents the *Arabidopsis ft-10* mutant, EV/*ft-10* represents overexpressed empty vector C15 in *ft-10* mutant, *SiHd3a*/*ft-10* represents overexpressed *SiHd3a* in *ft-10* mutant. Values followed by different letters in a column are significantly different at P ≤ 0.05 according to Duncan’s multiple range tests. Bars = 1 cm.

**Figure 7:**
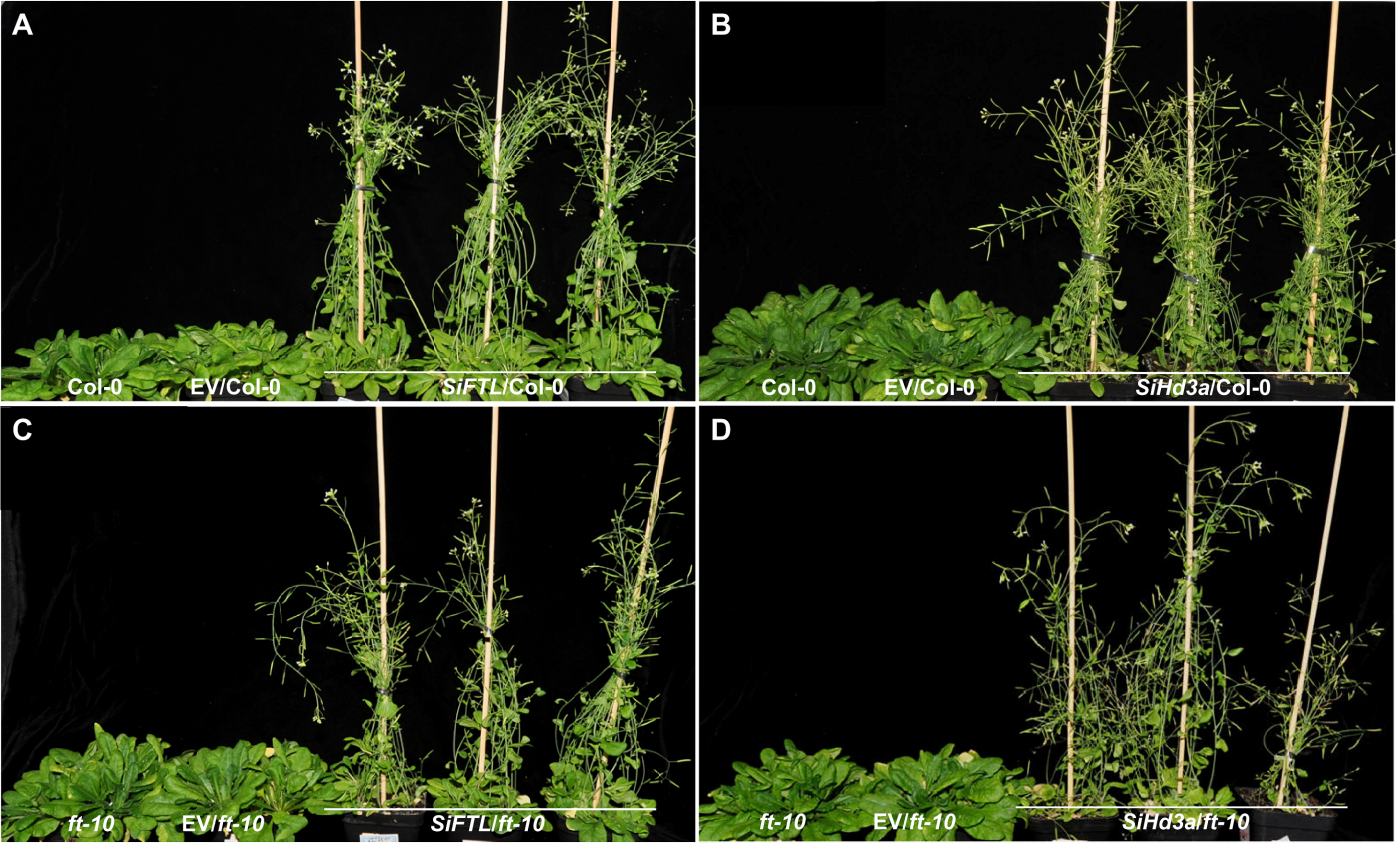
Days to flowering of transgenic *Arabidopsis* with overexpressed *SiFTL* or *SiHd3a* under SD condition. **(A)** Photographs of 50 d after sowing Col-0 and *SiFTL* transgenic lines. **(B)** Photographs of 50 d after sowing Col-0 and *SiHd3a* transgenic lines. **(C)** Photographs of 57 d after sowing *ft-10* mutant and *SiFTL* transgenic lines. **(D)** Photographs of 57 d after sowing *ft-10* mutant and *SiHd3a* transgenic lines.

The full-length coding sequences *SiFTL* and *SiHd3a* were amplified from cDNA using primers *SiFTL*-F/R and *SiHd3a*-F/R. To generate the recombination vector, *SiFTL* and *SiHd3a* were amplified using specific primers and then subcloned into the *BamHI* site of the C15 vector. The C15 vector was driven by the cauliflower mosaic virus 35S promoter (CaMV 35S) and contained a yellow fluorescent protein (YFP) epitope tag (Liu et al., 2019). Transgenic lines for *SiFTL* and *SiHd3a* were generated according to *Agrobacterium*-mediated transformation protocols. The seeds of the Col-0 ecotype were maintained in our laboratory, and seeds of the *Arabidopsis ft-10* mutant were kindly provided by Associate Researcher Zhang Xiaomei at the Institute of Crop Sciences, Chinese Academy of Agricultural Sciences. Col-0, *Arabidopsis ft-10* mutant, and transgenic plants were grown in a growth chamber under LD conditions (16 h light/8 h dark, 22/18°C) (**Figures 5**, **6**) or SD conditions (10 h light/14 hdark, 22/18°C) (**Figure 7**). We generated T_2_ transgenic lines and selected 30 representative overexpression (OE) lines for phenotypic analysis.

### Statistical analysis

Statistical analysis was conducted using JASP 0.95.1 software, with Duncan’s multiple range test at the 5% probability level (**Figures 1**, **2**, **5**, **6**). The heatmap was created with TBtools with row scaling and row clustering (Chen et al., 2020).

## Results

### Variations of flowering time in multiple populations

To identify late-flowering varieties of sesame, we conducted phenotypic identification of flowering time on more than 6,000 germplasm accessions and selected BS377 in the field, which was significantly delayed with longer photoperiods, making this an ideal genotype for studying flowering time. To map the candidate genomic region controlling flowering time, we developed several segregating populations by crossing Yuzhi 4 with BS377 (**Figure 1A**). Flowering time was evaluated in the SY environment using 150 BC_1_ accessions, 146 F_2_ accessions, and 128 F_2:3_ accessions. Additionally, flowering time was assessed for 150 BC_1_F_2_ accessions grown in three different locations LH, PY, and NY in 2018 (**Figure 1B**; **Supplementary Table S1**). The BC_1_, F_2_, and F_2:3_ populations grown in SY (SD conditions) exhibited more centralized and earlier flowering than the BC_1_F_2_ population grown under LD conditions (**Figure 1B**; **Supplementary Table S1**).

### QTLs for flowering time in sesame

The genetic linkage maps for BC_1_ and F_2_ populations were provided by Mei et al. (2023). By aligning the anchored SSR markers, the 14 LGs of the F_2_ map were integrated with the high-density BC_1_ map. A total of 152 markers were shared between the two maps, and the marker orders were highly consistent, demonstrating the robustness of the genetic maps (**Figure 1C**).

To identify candidate QTL regions controlling flowering time, we performed linkage mapping analysis using four populations (BC_1_, BC_1_F_2_, F_2_, and F_2:3_) grown in four distinct locations (SY, LH, PY, and NY) (**Figure 1C**; **Supplementary Table S4**). In these analyses, we detected two QTLs (*qFT_LG08–1* and *qFT_LG12-1*) in the F_2_ population, four QTLs (*qFT_LG01-1*, *qFT_LG01-4*, *qFT_LG08-4*, and *qFT_LG12-2*) in the F_2:3_ population, four QTLs (*qFT_LG01-2*, *qFT_LG01-3*, *qFT_LG08-2*, and *qFT_LG12-3*) in the BC_1_ population, and six QTLs (*qFT_LG01-5*, *qFT_LG05-1*, *qFT_LG05-2*, *qFT_LG05-3*, *qFT_LG08-3*, and *qFT_LG13-1*) in the BC_1_F_2_ population.

It is worth noting that several QTLs were identified: *qFT_LG01-1*, *-2*, and *-3*, located closely on LG1, with PVE ranging from 7.1% to 28.1%, were exclusively detected under SD condition; *qFT_LG05-1*, *-2*, and *-3*, clustered on LG5, with PVE from 7.1% to 9.3%, were only found under LD condition; *qFT_LG08-1*, *-2*, *-3*, and *-4*, situated near each other on LG8, with PVE from 9.5% to 57.6%, were detected under both SD and LD conditions; and *qFT_LG12-1*, *-2*, and *-3*, closely positioned on LG12, with PVE from 2.6% to 17.2%, were identified solely under SD condition.

A total of 16 QTLs were detected in the F_2_ and BC_1_ population under the six environments (**Figure 1C**; **Supplementary Table S4**). There were four QTL loci (*qFT_LG08-1*,*-2*, *-3*, and*-4*), which were stable (detected in different environments and/or populations) and with large effect (with a PVE > 9%). These QTLs were consistently detected under both LD and SD conditions, explaining 9.5% to 57.6% of the PVE. By aligning the markers to the sesame reference genome Yuzhi 4, we found that the *qFT_LG08-1*, *-2*, *-3*, and *-4* regions overlapped, pinpointing a 13.54-13.95 Mb region (~ 400 kb) on chromosome 11 as the primary candidate region responsible for flowering time.

### RNA-seq and qRT-PCR analyses validate candidate genes regulating sesame flowering time

To further identify the candidate genes regulating flowering time, we analyzed the expression profiles of genes within the candidate region (~ 400 kb on chromosome 11) across eight developmental stages in both Yuzhi 4 and BS377 (**Figure 2**; **Supplementary Figure 1**; **Supplementary Table S5**). After removing adapters and filtering out low-quality and short reads, the clean reads were mapped to the unpublished Yuzhi 4 genome. Within this region, 63 genes were identified for further analysis.

KEGG enrichment analysis highlighted that the “Circadian rhythm-plant” pathway, which includes the genes *Sin05626* and *Sin05627*, is associated with flowering time regulation (**Figure 2A**; **Supplementary Table S5**). GO enrichment analysis further supported this, revealing terms related to the “vegetative to reproductive phase transition of the meristem” suggesting potential roles in flowering time control (**Supplementary Figure 1**; **Supplementary Table S5**). Among the candidate genes, *Sin05626* and *Sin05627* were prioritized as key players in this process. Gene annotation indicated that *Sin05626* and *Sin05627* encode FLOWERING LOCUS T-like (*SiFTL*) and HEADING DATE 3A (*SiHd3a*) proteins, respectively, suggesting their potential as central regulators of flowering time in sesame. The two genes are located adjacent to each other on chromosome 11, separated by only 2.2 kb (**Supplementary Table S5**).

Expression profiling showed that *SiFTL* and *SiHd3a*, among the 63 candidate genes, exhibited expression patterns positively correlated with flowering time (**Figure 2B**; **Supplementary Figure 1**). In Yuzhi 4, the expression level of *SiFTL* increased with flowering formation process (**Figure 2B**; **Supplementary Figure 1**). Notably, *SiFTL* expression was undetectable in BS377 across all developmental stages, suggesting that its loss of expression contributes to the late-flowering phenotype. In contrast, the expression of *SiHd3a* increased during flowering progression in both Yuzhi 4 and BS377 (**Figure 2C**; **Supplementary Figure 1**). To validate these findings, qRT-PCR analysis was performed to assess the expression of candidate genes across eight developmental stages in Yuzhi 4 and BS377. The results confirmed the expression patterns observed in the RNA-seq data, thereby supporting the reliability of the RNA-seq analysis (**Figure 2**).

### Gene structure and phylogenetic analysis of *SiFTL* and *SiHd3a*

To investigate the genetic basis of flowering time variation, we analyzed the genomic sequences of *SiFTL* and *SiHd3a* in both Yuzhi 4 and BS377 (**Figure 3A**). In Yuzhi 4, the *SiFTL* gene comprises five exons, with its first exon and intron spanning 17 bp and 2,094 bp, respectively. A major structural variation was identified in BS377: a 6,265 bp insertion within the first intron of *SiFTL*, which harbors several conserved domains for transposase and nuclease. While seven SNPs were found in the exonic regions (**Supplementary Figure 2**), no significant variations were found in the promoter region. The complete absence of *SiFTL* expression across all examined stages in BS377 suggests that this large insertion may be responsible for its silencing. For *SiHd3a*, sequence compassion revealed only three exonic SNPs in BS377 (**Supplementary Figure 2**), with no notable changes in the promoter region.

To elucidate the evolutionary relationships of these genes, we constructed a phylogenetic tree using 57 orthologs from *Arabidopsis*, rice, soybean, and sesame (**Figure 3B**; **Supplementary Table S6**). We identified 11 PEBP genes in sesame, which were divided into three subfamilies: *FT-like* (4 genes), *TFL1-like* (5 genes), and *MFT-like* (2 genes). The *FT-lik*e subfamily includes one *SiFTL* (*Sin05626*), two *Hd3a* genes (*Sin05627* and *Sin22335*), and one *Hd3a-like* gene (*Sin08147*). Notably, both *SiFTL* and *SiHd3a* (*Sin05627*) were located within the previously identified ~ 400 kb region on chromosome 11. Phylogenetically, *SiFTL* and *SiHd3a* cluster closely with the known floral promoters *AtFT* and *AtTSF* from *Arabidopsis*, suggesting their role as key regulators of flowering in sesame.

We further validated their roles through haplotype analysis in the parental lines and the RIL population (**Figure 4**). Presence of the *SiFTL* BS377 haplotype band correlated with late flowering, while presence of the *SiHd3a* Yuzhi 4 haplotype band correlated with early flowering. The results clearly showed that the BS377 haplotype of both *SiFTL* and *SiHd3a* were consistently associated with late flowering, whereas the Yuzhi 4 haplotype co-segregated with early flowering phenotype.

### Heterologous expression of *SiFTL* and *SiHd3a* promotes flowering in *Arabidopsis*

To functionally validate their roles, we expressed the Yuzhi 4 haplotype of *SiFTL* and *SiHd3a* in both wild-type *Arabidopsis* Col-0 and the late-flowering mutation *ft-10* (**Figure 5**). Under LD condition, heterologous expression of *SiFTL* significantly accelerated flowering. In the wild-type Col-0 background, *SiFTL* overexpression lines flowered in 19.1 days with 4.8 rosette leaves, markedly earlier than the controls (32.5 and 32.1 days for Col-0 and the empty vector (EV)/Col-0; 17.3 and 17.9 rosette leaves for Col-0 and EV/Col-0). The OE-*SiFTL* lines exhibited significantly earlier flowering compared to the controls (**Figures 5A–C**, **G, H**), demonstrating that *SiFTL* can promote flowering in *Arabidopsis*. In the *ft-10* mutant, the flowering times were 46.0 days for *ft-10*, 45.1 days for EV/*ft-10*, and 18.4 days for *SiFTL*/*ft-10*. The rosette leaf numbers were 32.1 for *ft-10*, 31.7 for EV/*ft-10*, and 5.1 for *SiFTL*/*ft-10*. While the *ft-10* mutant displayed delayed flowering, heterologous expression of *SiFTL* significantly accelerated flowering (**Figures 5D–H**), further confirming that *SiFTL* plays a critical role in regulating flowering time.

To explore the function of *SiHd3a* in flowering under LD condition, we cloned *SiHd3a* into the C15 vector and transformed it into *Arabidopsis* Col-0 and the *ft-10* mutant (**Figure 6**). In wild-type Col-0, the flowering times were 32.5 days for Col-0, 32.1 days for EV/Col-0, and 16.1 days for *SiHd3a*/Col-0. The rosette leaf numbers were 17.3 for Col-0, 17.9 for EV/Col-0, and 3.9 for *SiHd3a*/Col-0. The *SiHd3a* lines showed significantly earlier flowering compared to the controls (**Figures 6A–C**, **G, H**), indicating that *SiHd3a* also plays a key role in flowering regulation. In the *ft-10* mutant, the flowering times were 46.0 days for *ft-10*, 45.1 days for EV/*ft-10*, and 16.9 days for *SiHd3a*/*ft-10*. The rosette leaf numbers were 32.1 for *ft-10*, 31.7 for EV/*ft-10*, and 4.7 for *SiHd3a*/*ft-10*. Similar to *SiFTL*, heterologous expression of *SiHd3a* in the *ft-10* mutant significantly rescued the delayed flowering phenotype (**Figures 6D–H**), further supporting the role of *SiHd3a* as a key regulator of flowering time in *Arabidopsis*.

We also tested the OE lines for phenotypic analysis under SD condition (**Figure 7**). In the *SiFTL*/Col-0, *SiFTL*/*ft-10*, *SiHd3a*/Col-0, *SiHd3a*/*ft-10* lines, the flowering times were 23.1 d, 19.5 d, 18.5 d, and 18.9 d, respectively. The corresponding rosette leaf numbers were 6.4, 5.2, 4.5, and 4.3. By 57 d, Col-0, EV/Col-0, *ft-10*, and EV/*ft-10* had not developed inflorescences. These results indicated that overexpression of *SiFTL* and *SiHd3a* promotes flowering under SD conditions, demonstrating that *SiFTL* and *SiHd3a* play key roles in flowering regulation.

## Discussion

Flowering time is crucial for crop adaptation and yield, which also impacts biomass and productivity. The photoperiod regulatory pathway for flowering time has been identified in many plants, including *Arabidopsis* (Wang et al., 2024), rice (Komiya et al., 2008), soybean (Lin et al., 2021), sorghum (Tadesse et al., 2024) and others (**Supplementary Table S6**). However, sesame flowering is promoted under SD conditions, and it is therefore classified as a SD crop (Zhou et al., 2018). Although some key genes related to flowering time, such as *SiCOL1*, *SiFT*, *SiFT4*, and *SiFTL*, have been identified (Zhou et al., 2018; López et al., 2023; Sabag et al., 2024), the photoperiod regulation mechanism of sesame flowering time has not yet been reported. In our study, molecular function, gene expression, sequence variations, and transgenic analysis of the key candidate genes in sesame controlling flowering time were comprehensively analyzed.

Genetic resources are important for genetic improvement and crop breeding. The late-flowering material BS377, with late flowering under SD conditions and no flowering in LD conditions, is an ideal system to study flowering time in sesame breeding. Genetic analysis across multiple segregating populations (BC_1_, F_2_, F_2:3_, and BC_1_F_2_) derived from crosses between the late-flowering variety ‘BS377’ and the variety ‘Yuzhi 4’ has pinpointed that ~ 400 kb overlapping region on chromosome 11 that exhibits the strongest and most consistent effects on flowering time (**Figure 1**; **Supplementary Table S4**). This finding refines the previously reported 1.3 Mb interval on linkage group 11 associated with flowering time under optimal and late sowing condition (Sabag et al., 2024).

Phylogenetic analysis can provide valuable insights into evolutionary relationships and the potential function of candidate genes. Phylogenetic analysis revealed that *SiFTL*, *SiHd3a*, *Sin08147*, and *Sin22335* cluster within the *FT-like* subfamily and are closely related to *AtFT*, *AtTSF*, *OsRFT1* and *OsHd3a* (**Figure 3B**). These genes are well-known florigens that induce flowering in response to photoperiod and circadian rhythms (Komiya et al., 2008; Jin et al., 2015; Colleoni et al., 2024; Wang et al., 2024). Since *Sin08147* and *Sin22335* were not located in the candidate region, we infer that *Sin08147* and *Sin22335* are not important in regulating sesame flowering time.

In the candidate region, by combining the transcript expression levels and sequence alignment of the two parental lines, *SiFTL* and *SiHd3a* were found to be positively correlated with flowering time (**Figure 2**; **Supplementary Figure 1**). RNA-seq and qRT-PCR analyses revealed that the expression levels of *SiFTL* and *SiHd3a* in Yuzhi 4 exhibited a continuous upregulation during flowering progression. These expression profiles were consistent with previous reports indicating that these candidate genes are highly expressed during flowering (Komiya et al., 2008; Sabag et al., 2024). Recent studies have investigated *SiFTL* and *SiHd3a* genes regulating flowering (López et al., 2023; Sabag et al., 2024). However, the expression profile of *SiFTL* diverged from prior findings. Previous results indicated high expression of *SiFTL* in K3 and NEB varieties following floral induction (López et al., 2023). Conversely, *SiHd3a* does not appear to regulate the transition from vegetative to reproductive growth in K3 and NEB varieties. Additionally, no significant differences in its expression levels were observed between the early-flowering S-490 and late-flowering S-10 genotypes. These findings suggest that *SiFTL* and *SiHd3a* may not be primary flowering candidates, likely due to the limited growth stages analyzed in earlier studies (López et al., 2023; Sabag et al., 2024). In our study, *SiFTL* and *SiHd3a* were identified as candidate genes through analyzing their expression patterns across eight developmental stages.

Comparative genomic alignment revealed a 6,265 bp insertion within the first intron of *SiFTL* in BS377, a structural alteration that is likely to disrupt proper transcript stability and thereby contribute to the observed silencing (**Figures 2**, **3**; **Supplementary Figure 1**). This finding parallels the *ft-10* mutant in *Arabidopsis*, in which a T-DNA insertion in the first intron of *AtFT* abolishes *FT* expression and confers pronounced late flowering (Liu et al., 2014). Collectively, these data indicate that the transcriptional silencing of *SiFTL* in BS377 is the primary cause of its delayed flowering. Although previous studies suggest that the C/T allele in *SiFTL* contributes to flowering time regulation (Sabag et al., 2024), this study reveals that the structure of *SiFTL* in BS377 differs from that in S-490 and S-10, and plays a pivotal role as a key determinant of flowering time. For *SiHd3a*, three SNPs were detected in the exonic regions of BS377 compared to the Yuzhi 4, with no significant variations observed in its promoter region. In our study, *SiFTL* and *SiHd3a* cluster within the *FT-like* subfamily and are tightly clustered on chromosome 11, separated by only 2.2 kb (**Supplementary Table S5**). This aligns with prior findings that these genes are physically linked (Sabag et al., 2024). In the rice system, where *RICE FLOWERING LOCUS T 1* (*RFT1*) and *OsHd3a* are closely linked (11.5 kb apart) on chromosome 6 (Komiya et al., 2008), this finding provides clues that *SiFTL* and *SiHd3a* are important genes for flowering time.

Based on these sequence differences, molecular markers of *SiFTL* and *SiHd3a* were designed and validated in a RIL population (**Figure 4**). Based on RIL population analysis, we found that *SiFTL* and *SiHd3a* genes were closely linked and showed complete co-segregation with flowering time. RIL population resequencing analysis suggests that this locus, rather than the two genes themselves, is associated with sesame flowering time (**Supplementary Figure 4**). However, to determine whether *SiFTL* and *SiHd3a* are key candidate genes for regulating flowering time in sesame, each gene’s needs to be verified using gene editing technology. In addition, the specific primers designed for *SiFTL* and *SiHd3a* can be used as molecular markers to assist in the selective breeding of sesame at the flowering stage, or provide valuable genetic resources for breeding early maturing sesame varieties.

Heterologous expression of *SiFTL* and *SiHd3a* in *Arabidopsis* significantly accelerated flowering under LD and SD conditions (**Figures 5**–**7**). However, at 57 d under SD conditions, Col-0 and *ft-10* did not develop inflorescences. Moreover, the flowering time of *Arabidopsis* overexpressing *SiFTL* or *SiHd3a* did not differ significantly between LD and SD conditions. This indicates that *SiFTL* and *SiHd3a* act as inducers of flowering under SD conditions as well as LD conditions, suggesting they may function as universal floral activators downstream. This study further elucidates the regulatory roles of *SiFTL* and *SiHd3a* in sesame flowering through their heterologous overexpression in *Arabidopsis* Col-0 and *ft-10* mutant, building upon and extending the findings of Sabag et al. (2024).

## Conclusions

Flowering time is an important adaptive character that significantly influences plant biomass and adaptability. In this study, we pinpoint ~ 400 kb region on chromosome 11 that exhibits the strongest and most stable effect on flowering time. Transcript profiling revealed a steady increase in the expression of *SiFTL* and *SiHd3a* during flower development in Yuzhi 4. Integrative analyses of gene structure and expression data further demonstrated that the silencing of *SiFTL* transcripts in the variety BS377 underlies its markedly delayed flowering. Phylogenetic analysis suggested that *SiFTL* and *SiHd3a* are orthologues of *AtFT* and are associated with flowering in sesame. Overexpression of *SiFTL* and *SiHd3a* in *Arabidopsis* Col-0 and *ft-10* mutation accelerated flowering under both LD and SD conditions, suggesting they may function as universal floral activators downstream. Finally, in the cultivar Yuzhi 4, the *SiFTL* and *SiHd3a* locus is closely associated with precocity. Overall, our results demonstrate that *SiFTL* and *SiHd3a* serve as crucial regulators of flowering in sesame, providing valuable insights for the molecular breeding of early-maturing cultivars.

## Data Availability

The datasets presented in this study can be found in online repositories. The names of the repository/repositories and accession number(s) can be found in the article/**Supplementary Material**.

## Supplementary material

Supplementary Figure 1 Candidate genes identification through RNA-seq. **(A)** GO enrichment analysis for genes in the candidate regions. **(B)** Heatmap for genes in the candidate regions across eight developmental stages of Yuzhi 4 and BS377 new leaves. S1, S2, S3, S4, S5, S6, S7, S8 represent the leaves of the Yuzhi 4 variety at 5, 10, 15, 20, 25, 30, 35 and 40 days after sowing. S1, S2, S3, S4, S5, S6, S7, S8 represent the leaves of the BS377 variety at 5, 15, 35, 60, 65, 70, 75, and 90 d after sowing. S6 and S7 indicate the pre-budding and post-budding stages, respectively, while S8 denotes the post-flowering stage.

Supplementary Figure 2 Sequence alignment and variation analysis of *Sin05626* and *Sin05627* genes in Yuzhi 4 and BS377. **(A)** Sequence alignment of *SiFTL* (*Sin05626*) between BS377 and Yuzhi 4. **(B)** Sequence alignment of *SiHd3a* (*Sin05627*) between BS377 and Yuzhi 4.

Supplementary Figure 3 Schematic of primer design for genotyping *SiFTL* and *SiHd3a* loci in the RIL population. **(A)** Primer design of *SiFTL* in Yuzhi 4 and BS377. **(B)**: Primer design of *SiHd3a* in Yuzhi 4 and BS377. Pink represents insert sequence; Yellow represents primer; Green represents same sequence.

Supplementary Figure 4 Haplotype composition at the *SiFTL* and *SiHd3a* loci across the RIL population. **(A)** Genotype heat map of candidate interval. **(B)** Comparative analysis of flowering time of two haplotypes. Haplotype 1 (HAP1) represents the BS377 haplotypes at the *SiFTL* and *SiHd3a* loci. HAP2 represents the Yuzhi 4 haplotypes at the *SiFTL* and *SiHd3a* loci.
