# Supplementary figures and images for: SiFTL and SiHd3a are positive regulators of flowering time in sesame (Sesamum indicum L.)
Source: Front Plant Sci. 2025 Dec 3;16:1716212. doi: 10.3389/fpls.2025.1716212 (PMC12708595; doi:10.3389/fpls.2025.1716212)

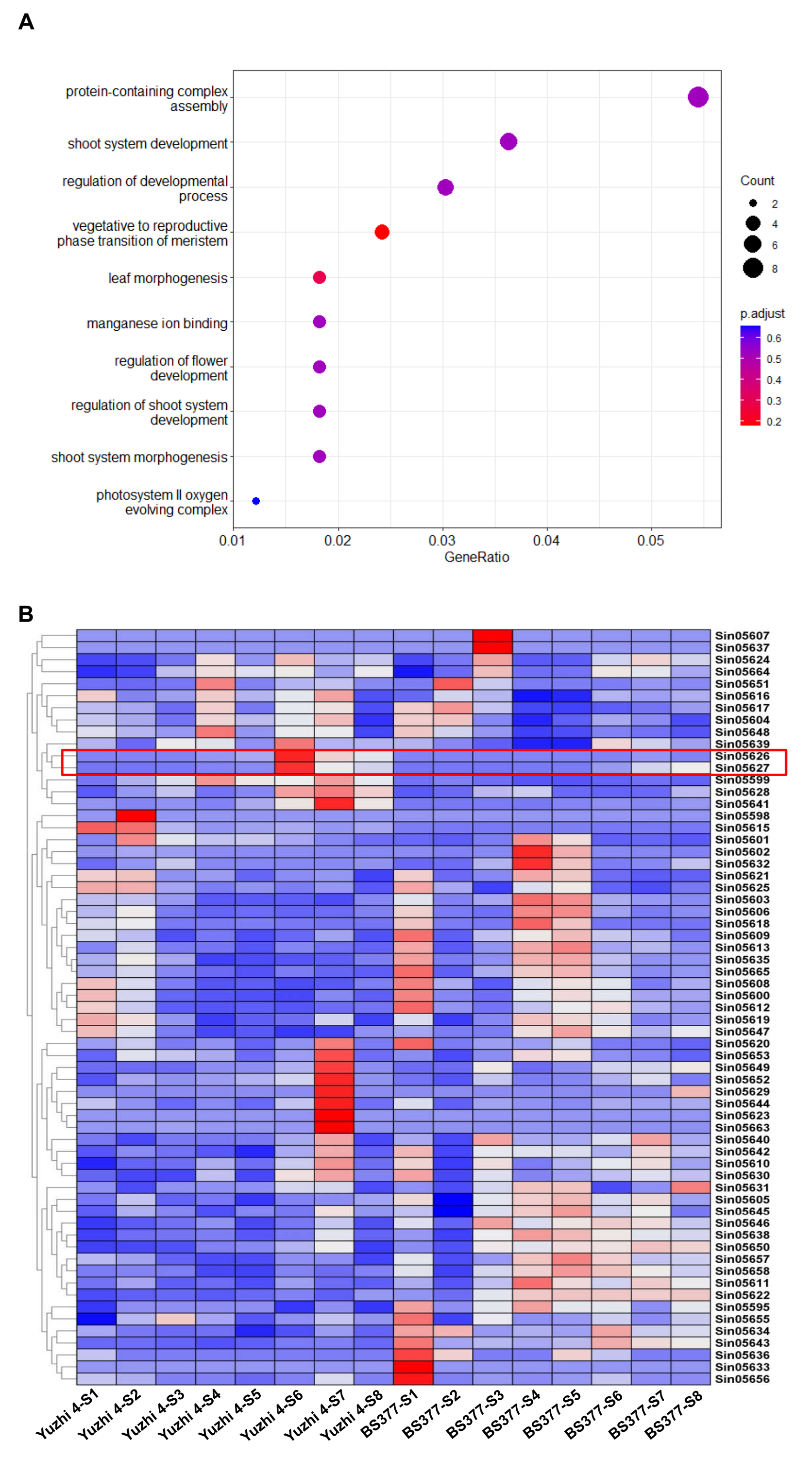

Supplement: Supplementary Figure 1 — Candidate genes identification through RNA-seq. (A) GO enrichment analysis for genes in the candidate regions. (B) Heatmap for genes in the candidate regions across eight developmental stages of Yuzhi 4 and BS377 new leaves. S1, S2, S3, S4, S5, S6, S7, S8 represent the leaves of the Yuzhi 4 variety at 5, 10, 15, 20, 25, 30, 35 and 40 days after sowing. S1, S2, S3, S4, S5, S6, S7, S8 represent the leaves of the BS377 variety at 5, 15, 35, 60, 65, 70, 75, and 90 d after sowing. S6 and S7 indicate the pre-budding and post-budding stages, respectively, while S8 denotes the post-flowering stage. [file Image1.tif]

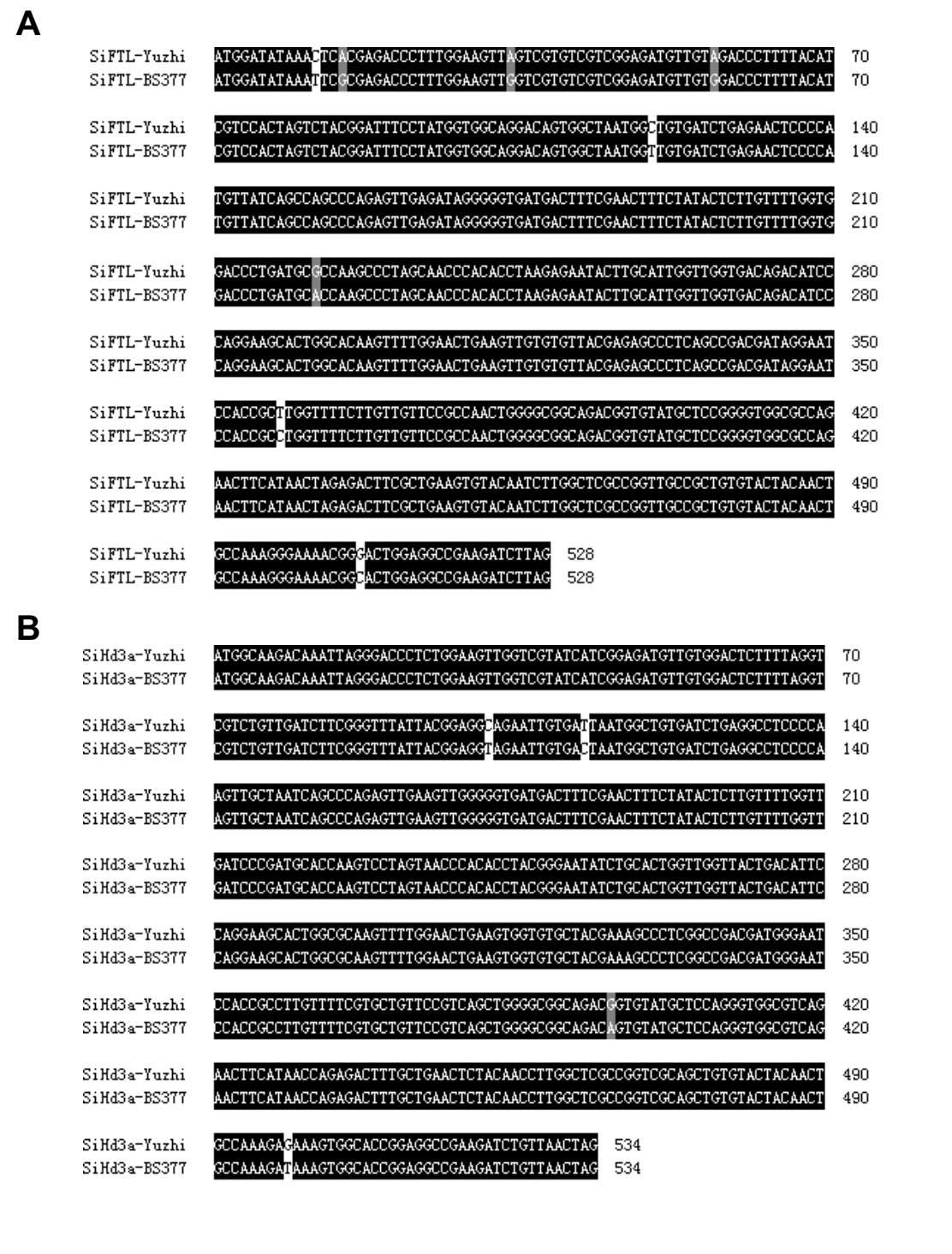

Supplement: Supplementary Figure 2 — Sequence alignment and variation analysis of Sin05626 and Sin05627 genes in Yuzhi 4 and BS377. (A) Sequence alignment of SiFTL (Sin05626) between BS377 and Yuzhi 4. (B) Sequence alignment of SiHd3a (Sin05627) between BS377 and Yuzhi 4. [file Image2.tif]

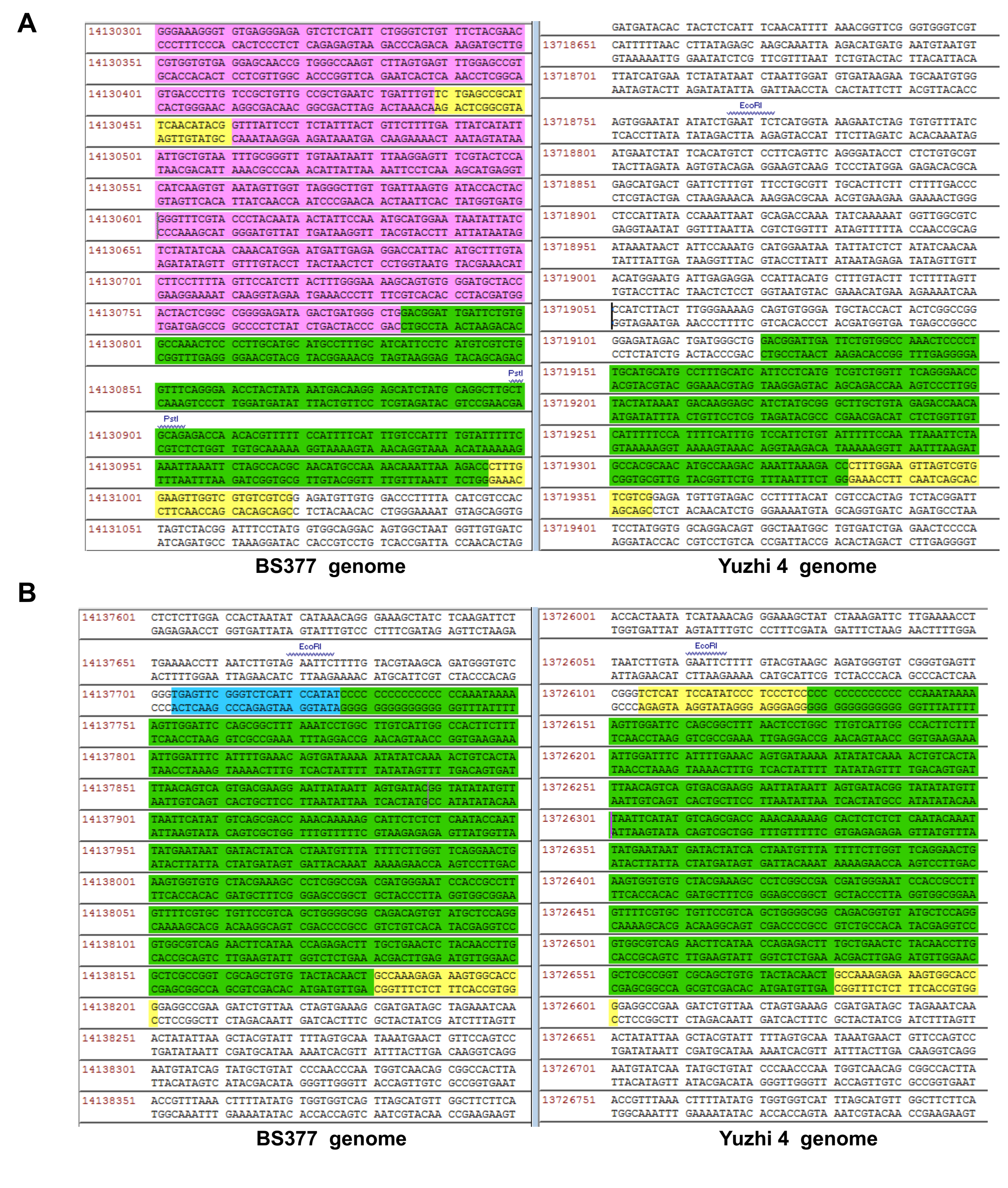

Supplement: Supplementary Figure 3 — Schematic of primer design for genotyping SiFTL and SiHd3a loci in the RIL population. (A) Primer design of SiFTL in Yuzhi 4 and BS377. (B): Primer design of SiHd3a in Yuzhi 4 and BS377. Pink represents insert sequence; Yellow represents primer; Green represents same sequence. [file Image3.tif]

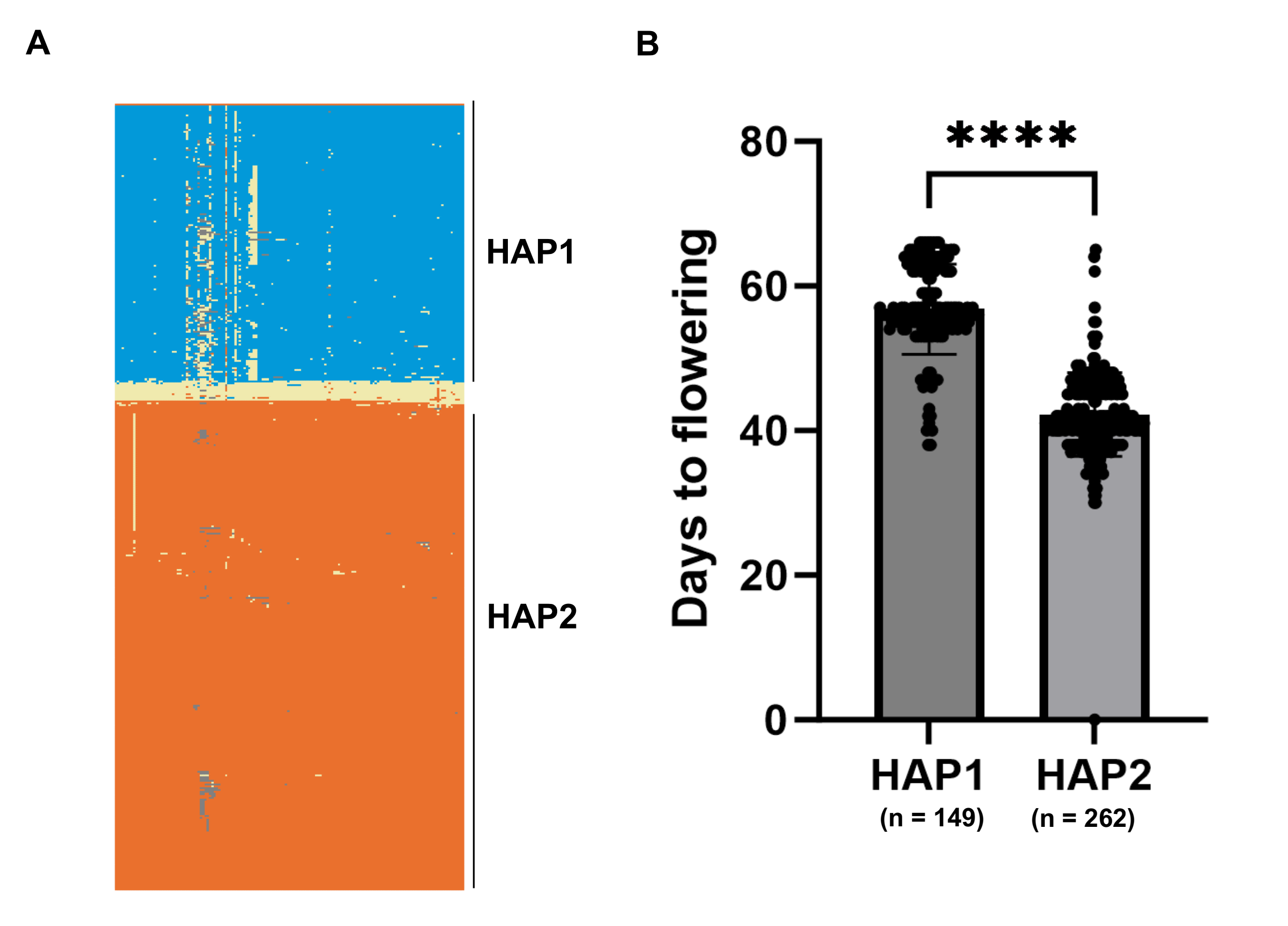

Supplement: Supplementary Figure 4 — Haplotype composition at the SiFTL and SiHd3a loci across the RIL population. (A) Genotype heat map of candidate interval. (B) Comparative analysis of flowering time of two haplotypes. Haplotype 1 (HAP1) represents the BS377 haplotypes at the SiFTL and SiHd3a loci. HAP2 represents the Yuzhi 4 haplotypes at the SiFTL and SiHd3a loci. [file Image4.tif]
